# Cardiovascular disease risk profile and management among people 40 years of age and above in Bo, Sierra Leone: A cross-sectional study

**DOI:** 10.1371/journal.pone.0274242

**Published:** 2022-09-09

**Authors:** Maria Lisa Odland, Khadija Gassama, Tahir Bockarie, Haja Wurie, Rashid Ansumana, Miles D. Witham, Oyinlola Oyebode, Lisa R. Hirschhorn, Justine I. Davies

**Affiliations:** 1 Institute of Applied Health Research, University of Birmingham, Edgbaston, Birmingham, United Kingdom; 2 Department of Obstetrics and Gynecology, St Olav’s Hospital, Trondheim University Hospital, Trondheim, Norway; 3 Malawi-Liverpool-Wellcome Trust Research Institute, Blantyre, Malawi; 4 Institute of Life Course and Medical Sciences, University of Liverpool, Liverpool, United Kingdom; 5 Department of Public Health and Nursing, Norwegian University of Science and Technology, Trondheim, Norway; 6 Warwick Medical School, University of Warwick, Coventry, United Kingdom; 7 College of Medicine and Allied Health Sciences, University of Sierra Leone, Freetown, Sierra Leone; 8 School of Community Health Sciences, Njala University, Bo Campus, Bo, Sierra Leone; 9 AGE Research Group, NIHR Newcastle Biomedical Research Centre, Newcastle University and Newcastle upon Tyne Hospitals NHS Foundation Trust, Newcastle, United Kingdom; 10 Department of Medical Social Sciences, Northwestern University Feinberg School of Medicine, Chicago, IL, United States of America; Centers for Disease Control and Prevention, UNITED STATES

## Abstract

**Introduction:**

Access to care for cardiovascular disease risk factors (CVDRFs) in low- and middle-income countries is limited. We aimed to describe the need and access to care for people with CVDRF and the preparedness of the health system to treat these in Bo, Sierra Leone.

**Methods:**

Data from a 2018 household survey conducted in Bo, Sierra Leone, was analysed. Demographic, anthropometric and clinical data on CVDRF (hypertension, diabetes mellitus or dyslipidaemia) from randomly sampled individuals 40 years of age and above were collected. Future risk of CVD was calculated using the World Health Organisation–International Society of Hypertension (WHO-ISH) calculator with high risk defined as >20% risk over 10 years. Requirement for treatment was based on WHO package of essential non-communicable (PEN) disease guidelines (which use a risk-based approach) or requiring treatment for individual CVDRF; whether participants were on treatment was used to determine whether care needs were met. Multivariable regression was used to test associations between individual characteristics and outcomes. Data from the most recent WHO Service Availability and Readiness Assessment (SARA) were used to create a score reflecting health system preparedness to treat CVDRF, and compared to that for HIV.

**Results:**

2071 individual participants were included. Most participants (n = 1715 [94.0%]) had low CVD risk; 423 (20.6%) and 431 (52.3%) required treatment based upon WHO PEN guidelines or individual CVDRF, respectively. Sixty-eight (15.8%) had met-need for treatment determined by WHO guidelines, whilst 84 (19.3%) for individual CVDRF. Living in urban areas, having education, being older, single/widowed/divorced, or wealthy were independently associated with met need. Overall facility readiness scores for CVD/CVDRF care for all facilities in Bo district was 16.8%, compared to 41% for HIV.

**Conclusion:**

The number of people who require treatment for CVDRF in Sierra Leone is substantially lower based on WHO guidelines compared to CVDRF. CVDRF care needs are not met equitably, and facility readiness to provide care is low.

## Introduction

The prevalence of cardiovascular disease (CVD—for example, myocardial infarction and strokes) and its risk factors (CVDRF, for example, diabetes, hypertension, or hypercholesterolaemia) is increasing in lower- and -middle income countries (LMICs) [1]. However, access to high-quality care for these conditions in many LMICs is known to be limited, resulting in suboptimal metrics at each point in the cascade of care—being diagnosed, receiving treatment, and having the condition controlled [2–5].

CVD and CVDRF are chronic conditions, with potentially complex management requirements, and the necessity for ongoing, potentially multidisciplinary, care. Hence, a healthcare platform consisting of coordinated, continuous, and comprehensive health system structures and processes ideally needs to be in place to provide high quality outcomes [6–8]. Although, it is recognised that the availability of structures and processes (often considered as healthcare service readiness) doesn’t necessarily equate to receipt of effective clinical care [9, 10], healthcare service readiness is a necessary requirement for this [9]. While more data are needed, studies available suggest that health service readiness for providing effective diagnosis and clinical care for CVD and CVDRF is limited in many LMICs [11–13].

Scaling up healthcare services to provide for CVDRFs is cost-effective, with treating individual CVDRFs to target known to be less cost effective than using a risk-based approach with treatment for CVDRF given based upon the individual’s future predicted risk of CVD [14–16]. International guidelines developed for use in LMICs, such as WHO package of essential non-communicable (PEN) guidelines and South Africa’s PC 101, advocate a risk-based approach to managing CVDRFs. Determining which policies to implement for the management of CVDRF at a country level requires understanding of the current status of services, the population in need of treatment, and the population who is currently accessing services. In, Sierra Leone where the focus of healthcare provision has been on infectious diseases and maternal, neonatal and child health, this information is lacking.

In this analysis done in Bo, one of the 16 districts in Sierra Leone, we describe individual’s estimated CVD risk and socio-demographic factors associated with being at high risk; assess numbers of people who would require risk-based treatment based on current WHO PEN guidelines or a treat to target approach based on the presence of one or more individual CVDRFs; assess whether people who require treatment are accessing it; and show service readiness to provide care for CVD/CVDRF. Finally, we compare service readiness for CVD/CVDRF to that for HIV care which has been a main focus of funding during the millennium development goal (MDG) era, to give an indication of what level of service readiness is possible to achieve with investment.

## Methods

This is an analysis of the data from a 2018 household survey conducted in Bo, Sierra Leone and data from the most recent WHO Service Availability and Readiness Assessment (SARA) done in 2017 [2, 17].

### Setting

Sierra Leone is located in West Africa. It is one of the least developed countries in the world. In 2017, the percentage of the gross domestic product (GDP) spent on health was 8.75% [18]. However, domestic general government health expenditure is only 1.23% of GDP; out of pocket (OOP) expenditure contributes 55.18% of total health expenditure and the external funding of health is high [18, 19]. The focus of external funding for health has been on communicable diseases, whereas NCDs (of which CVD and CVDRF are only a subset) received only $510,000 of a total of $170 million in 2017 [20, 21]. By contrast, HIV services received $30 million [20, 21].

The study was carried out in Bo district in the Southern province of Sierra Leone. It is the fifth most populous district in the country and comprises 15 rural chiefdoms and 24 urban areas [22]. Its district headquarters, Bo, is the second largest city in Sierra Leone [22]. The district has a recorded population of 575,478 constituting 8.1% of the country’s population with the majority living in rural areas (66.1%) [22]. Adults aged 40 years of age and above, among whom this study was done, comprise 17.4% of the total population [22]. In Bo district, healthcare is provided by a mix of public and private–for profit or not for profit–facilities at the primary or secondary healthcare levels [22].

### Household survey

The study sample included were men or women 40 years of age and above, this age group was selected given the increasing risk of CVDRF with age and to be congruent with other similar surveys [2, 23–26]. The surveys were developed in English, but translated into Mende or Krio by a bilingual speak, and back translated into English to check the accuracy of the translation.

#### Sampling

Numbers of participants to sample from urban and rural areas were calculated based on the proportions of people known to be living in these areas. The population in the area was not well delineated in census data, therefore sampling proceeded by first randomly selecting from rural chiefdoms or urban sub districts and, for the rural areas, by further randomly selecting villages or settlements from each chiefdom. Seven rural chiefdoms or urban sub districts were randomly selected to participate, and two settlements or villages were further randomly selected within each rural chiefdom.

#### Data collection

Data were collected electronically by trained data collectors using ODK software. Survey questions asked gender, age, marital status (as single, cohabiting, currently married, multiple partners, divorced, widowed, or refused), and highest level of education completed (no formal schooling, primary, junior secondary, senior secondary, higher education, or refused). There were 49 questions on house construction materials and household assets. Questions on smoking, awareness of presence of CVD or CVDRF, and whether respondents were on treatment for these risk factors were based on the WHO Stepwise survey; for those who reported suffering from a CVDRF, whether care had been accessed was asked. Blood pressure was measured in the seated position using an Omron M6 AC LED Monitor. Three measurements were taken five minutes apart. Blood samples were taken in the morning after an 8 hour overnight fast. Glucose and cholesterol were measured using the Accutrend® Plus Blood Test Meter (Diagnostics Roche) point of care device, with cholesterol being measured in every second participant. If participants reported not fasting prior to blood sampling, they were recorded as non-fasting. The conversion rate of 1.11 was used to convert capillary glucose to plasma glucose [27]. Glucose was measured in all participants, whilst due to resource constraints, cholesterol samples were obtained from every second participant.

#### Definition of variables

Age was used as a continuous variable or categorised into the following groups: 40–49, 50–59, 60–69, 70–79, and >80. Educational level was dichotomised as any completed education (primary or higher) or no completed education. Marital status was categorised as single/widowed/divorced or married/cohabiting. Wealth quintiles were derived using Filmer and Pritchetts’ method from the first principal component of household assets and construction materials [28]. Based on thresholds for individual CVDRF in use at the time of the study, having hypertension was defined as systolic blood pressure ≥ 140 or diastolic ≥ 90mmHg, calculated using the average of the final two BP readings, or being on treatment for hypertension in the past two weeks. Diabetes was defined as fasting plasma glucose (FPG) ≥7.0 mmol/L (126 mg/dL), or random plasma glucose (RPG) ≥11.1 mmol/L (200 mg/dL), or being on treatment for diabetes in the past 2 weeks. Dyslipidaemia was defined as measured total cholesterol level ≥ 6.21 mmol/L, or low-density lipoprotein (LDL) ≥ 4.1 mmol/L, or high-density lipoprotein (HDL) < 1.19 mmol/L [2, 29, 30]. Smoking was classified as current smoker if participants either reported currently smoking or had ceased within in the last year, or non-smoking. Ten-year cardiovascular disease risk was computed using the WHO-ISH equations (which were in use at the time that the survey was done) using laboratory measurements [31]. WHO-ISH scores were derived from data collected on age, sex, smoking status, diabetes status as well as cholesterol and blood pressure measurements. Patients with self-reported MI, angina or stroke were excluded from the entire analysis as our consideration was primary prevention of CVD.

The methods have been described in full before [2].

### SARA survey

#### Data collection

Each of the 1284 public or private health facilities in Sierra Leone were included in the SARA survey [12]. The questionnaire was based upon the standard WHO SARA tool and the survey was conducted as detailed in their SARA report [17]. The questionnaires were pilot tested in facilities in the Western Area of Sierra Leone and adjusted before rolling out throughout the country. The SARA surveys capture data at each facility on presence of infrastructure and basic amenities, health workforce, service delivery and utilisation, availability of treatment guidelines, basic equipment, and treatments [12]. Data were collected by trained fieldworkers who administered the questionnaire to relevant facility staff. Data from facilities in Bo district were used for this study, to correspond to the population from which household survey data were collected.

#### Definition of variables

Facilities were classed as either primary or secondary care facilities (there are no tertiary hospitals in the district). Primary care clinics were Maternal and Child Health Posts (MCHP), Community Health Posts (CHP), and Community Health Centers (CHCs) [17]. Secondary care facilities were defined as district government hospitals and all other hospitals (private). Facility ownership was defined as government or private-owned. Facility readiness scores to provide care for CVDRF or HIV were derived from components required to deliver either HIV or CVDRF care. These were captured under the domains of equipment, diagnostic capacity, guidelines, medicines, and trained staff. Variables included in the initial survey were captured under the broad headings of non-communicable diseases or HIV, and as defined in the SARA manual [17, 32]. The authors further discussed variables to include or exclude from the above domains, based on clinical knowledge and experience of service provision in low resourced settings. What the different domains include is described in Appendix 1 in S1 File. For example MRI was not included as there is no MRI machine to be found in the country, and whilst it may be expected that a district level secondary hospital would have a CT scanner, this is not reasonable to expect of primary care facilities. Components included under each domain for CVDRF and HIV are shown in the S1 File. For both CVDRF and HIV, a score was created for each domain as the number of components present divided by the total number of components achievable. Scores are shown for each facility level by individual domain and inclusive of all domains.

### Outcome measures

The primary outcome measure was the proportion of the study sample defined as being at high 10-year risk of having a cardiovascular event, here defined as >20% risk as done in previous studies [33]. The WHO/ISH Risk score includes age, gender, smoking, diabetes, blood pressure, cholesterol and appropriate WHO epidemiological sub region [34]. Two secondary outcomes were studied–firstly the number of people who would require treatment for any CVDRF based on the WHO-PEN guidelines (which were available in 2018). WHO-PEN guidelines recommend treatment for hypertension if BP is ≥160/90mmHg or if BP is ≥140/90mmHg and CVD risk is >20%; treatment with hypoglycaemic agents is required if there is a diagnosis of diabetes; statin and aspirin treatment should be given if there is diabetes and a 10 year risk >20%, or 10 year risk is >30%; and ACE inhibitors should be given if diabetes is present and 10 year risk >20% (Appendix 2 in S1 File). The other secondary outcome was the number of people who would require treatment for the individual risk factors of diabetes, hypertension, or dyslipidaemia as defined by the study criteria, if a treat-to-target approach were used. Other outcomes describe access to care as the proportions of participants who required treatments under WHO-PEN guidelines and who were on those treatments and facility readiness to provide care.

### Sample size and statistical analysis

For the household survey, a sample size of 1893 participants was required to allow detection of diabetes prevalence (the risk factor thought likely to have the lowest prevalence) of 4% with a precision of ±1% [35]. To allow for non-response and non-availability of data, we oversampled by 20%. For the SARA survey, no sampling was done and all facilities in the district were included. WHO-risk scores were calculated using generated by the WHO/ISH Risk R-package [34].

For the household survey data, probability weights for age and sex in Bo were calculated from the 2015 Population and Household Census [22], and all analyses were done using weight adjustments. Continuous data are described as mean (SD) or median (IQR) if not normally distributed. Categorical variables are described as unweighted n and weighted %. For comparisons of continuous data we used t-tests or non-parametrics tests Mann-Whitney/ANOVA if data were skewed. Multivariable analyses of categorical outcomes were done using binary logistic regression. Age was not entered into the model assessing associations with high CVD risk, given its use in calculating the risk score. We did a complete case analysis for the multivariable analysis whilst the denominator for the univariate analysis varied. All analyses were done using SPSS V.26 (IBM).

### Ethical approval

Ethical approval was sought and given from the Sierra Leone Ethical and Scientific Review Committee and the BDM Research Ethics sub-committee at King’s College London (HR-17/18-7298). Consent to undertake the study was obtained from each village chief or community leader. Consent was obtained from all individuals participating in the study. In the events were participants were illiterate, the consent form was read out to them in the local language and an inked-thumb signature obtained.

## Results

**Fig 1:**
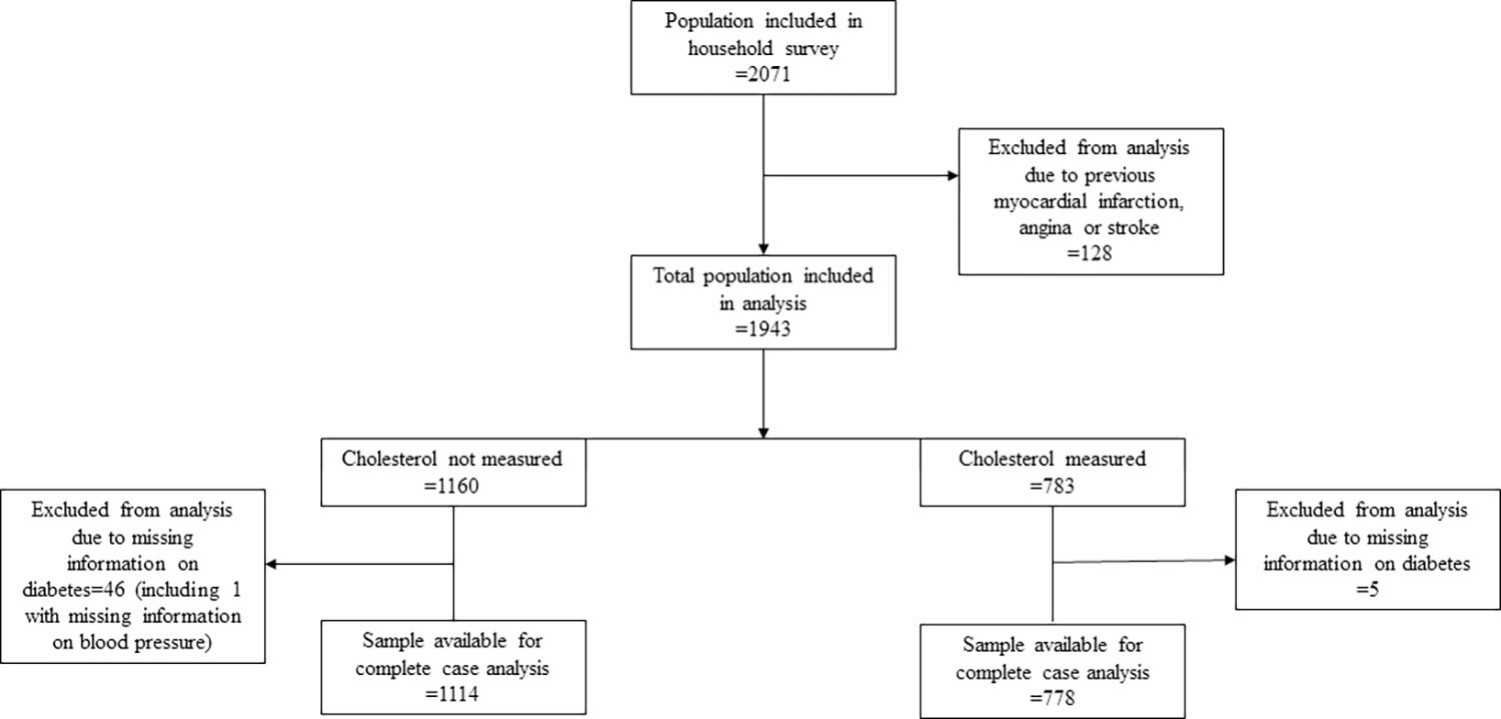
Flow diagram of individuals included in the study.

A total of 2071 individuals were included in the household survey study sample. The 128 participants who reported a prior history of myocardial infarction (n = 79), angina (n = 26) stroke (n = 29) were excluded leaving 1943 participants available for analysis (Fig 1). In total 778 participants (missing data = 5) had complete case information in the sample with measured cholesterol, and 1114 (missing data = 46) in the sample without cholesterol (Fig 1). Weighted and unweighted demographic characteristics of the study sample with and without cholesterol measured are presented in Table 1. Apart from age there were no differences between the two samples. In the total sample, the majority of the study sample was from rural areas 1167 (63.1%), there were slightly more males 845 (50.9%) than females and the mean age was 54.9 (SD 12.8) years. Most individuals were between 40–49 years old, 639 (44.0%), were married/cohabiting, 1297 (72.4%) and had no completed education 1306 (67.4%). Out of the sample that had cholesterol measured 431 (52.3%) had one or more CVDRFs (hypertension, diabetes, or dyslipidaemia defined using the study criteria).

**Table 1:** Unweighted and weighted demographic characteristics of the study sample in the sample with and without measured cholesterol.

		Sample with cholesterol measured (n = 778)		Sample without cholesterol measured (n = 1114)	
VARIABLE	VARIABLE GROUPS	Unweighted n (%)	Weighted (%)	Unweighted n (%)	Weighted (%)
LOCATION	Rural	484 (61.3)	63.3%	683 (61.3)	63.0%
	Urban	294 (38.7)	36.7%	431 (38.7)	37.0%
SEX	Female*	464 (59.6)	53.3%	583 (52.3)	46.1%
	Male	314 (40.4)	46.7%	531 (47.7)	53.9%
AGE	Mean (SD)	56.8 (13.1)	54.5 (12.5)	57.7 (13.4)	55.3 (13.05)
AGE GROUPS (5 missing)	40–49*	275 (35.5)	45.3%	364 (32.7)	43.0%
	50–59	213 (27.4)	24.9%	289 (26.0)	23.8%
	60–69	139 (17.9)	15.6%	208 (18.7)	16.2%
	70–79	85(10.9)	7.9%	160 (14.4)	10.4%
	≥80	64 (8.2)	6.3%	91 (8.2)	6.6%
MARITAL STATUS (2 missing)	Single / Divorced / Widowed*	248 (31.7)	27.5%	356 (30.7)	27.5%
	Married / Co-habiting	534 (68.3)	72.5%	803 (69.3)	72.5%
EDUCATION	No complete education	528 (67.9)	66.5%	778 (68.1)	68.8%
	Any education	250 (32.1)	33.5%	336 (31.9)	31.8%
WEALTH QUINTILES	1 (poorest)	137 (17.6)	18.3%	218 (20.5)	21.2%
	2	155 (19.9)	21.1%	216 (20.3)	20.8%
	3	139 (17.9)	18.7%	216 (20.3)	20.1%
	4	174 (22.4)	22.6%	192 (18.1)	18.1%
	5 (wealthiest)	152 (19.5)	19.3%	221 (20.8)	19.9%

*p<0.05 for comparison between the group which had cholesterol measurements done and the group that did not

### Primary outcome

Most participants (n = 1715 [94.0%]) had a risk score of ≤20% and were categorised as low risk. In binary logistic regression, including the following factors—area of living, educational level, marital status and wealth quintiles—living in urban areas, having no education, or being single/widowed/divorced was independently associated with higher CVD risk compared to the referent categories (Table 2).

**Table 2:** Multivariable associations between weighted demographic characteristics and being at high CVD risk (>20%) (n = 1892).

VARIABLE	GROUP	OR (95% CI)	p
Location	Rural	Referent	**-**
	Urban	1.15 (1.07–1.23)	<0.001
Educational level	No completed education	Referent	_
	Any education	0.90 (0.84–0.96)	0.002
Marital status	Married /co-habiting	Referent	_
	Single/divorced/widowed	2.50 (2.36–2.65)	<0.001
Wealth Quintiles	1 (poorest)	Referent	_
	2	0.71 (0.65–0.78)	< 0.001
	3	0.94 (0.86–1.02)	0.144
	4	0.89 (0.82–0.97)	0.011
	5 (wealthiest)	0.86 (0.78–0.95)	<0.001

### Secondary outcomes

According to WHO PEN guidelines, 423 (20.6%) of the study sample required treatment for at least one CVDRF (Table 3). When disaggregating by treatment required, 410 (20.0%) people required treatment for hypertension; 69 (3.3%) for diabetes; 74 (3.2%) for statin and/or aspirin; and 18 (0.6%) required an ACE-inhibitor (Table 4). When considering treat-to-target approaches for individual CVD risk factors, of the total sample, 431 (52.3%) would require treatment for at least one CVDRF. When disaggregating by condition requiring treatment 998 (48.4%) were hypertensive, 69 (3.3%) were diabetic, and 57 (6.9%) of those who had their cholesterol measured were dyslipidaemic.

**Table 3:** People that should be treated according to PEN guidelines or diagnostic criteria, and proportions on treatment currently n (weighted percentages) (n = 1892).

GROUPS	N (%*) of total sample that should be on any treatment according to WHO PEN guidelines	N (%*) of the sample who should be on treatment according to WHO PEN guidelines, and are receiving that treatment	N (%*) of total sample that should be on treatment of individual CVD risk factor (hypertension, or diabetes or cholesterol)	N (%*) on sample that should be on treatment for individual CVD risk factors and who are receiving that treatment
For any required CVDRF treatment	423 (20.6%)	68 (15.8%)	431 (52.3%)**	84 (19.3%)
For hypertension	410 (20.0%)	56 (13.4)	998 (48.4%)	136 (13.3%)
For diabetes	69 (3.3%)	13 (18.3)	69 (3.3%)	13 (18.3%)
With Statin/for dyslipidaemia	74 (3.2%)	0 (0)	57 (6.9%)**	0 (0%)**
With Aspirin	74 (3.2%)	5 (6.9%)		
With ACE-inhibitors	18 (0.6%)	No information		

Note that one participant may need multiple treatments so the total is lower than the sum of participants requiring treatment

*Weighted percentages

** Denominator n = 778 which is the sample with outcome variables including cholesterol measured

**Table 4:** Multivariable associations between weighted demographic characteristics and being on any treatment.

VARIABLE		Odds of being on treatment in those defined by PEN guidelines as requiring treatment		Odds of being on treatment in those who have a diagnosis of either hypertension, diabetes, or dyslipidemia*	
		OR (95% C.I)	p	OR (95% C.I)	p
Location	Rural	Referent		Referent	
	Urban	2.03 (1.82–2.26)	<0.001	1.30 (1.20–1.40)	<0.001
Gender	Female	Referent		Referent	
	Male	0.50 (0.44–0.59)	<0.001	1.03 (0.95–1.11)	0.516
Age group	40–49	Referent		Referent	
	50–59	2.17 (1.92–2.44)	<0.001	1.87 (1.71–2.04)	<0.001
	60–69	1.76 (1.54–2.02)	<0.001	2.89 (2.63–3.67)	<0.001
	70–79	1.79 (1.52–2.12)	<0.001	2.34 (2.06–2.65)	<0.001
	≥ 80	1.07 (0.87–1.31)	0.543	1.52 (1.29–1.79)	0.001
Educational level	No completed education	Referent		Referent	
	Any education	4.40 (3.90–4.83)	<0.001	2.56 (2.36–2.75)	<0.001
Marital status	Married / co-habiting	Referent		Referent	
	Single/divorced/widowed	1.06 (0.95–1.19)	0.288	2.08 (1.91–2.25)	<0.001
Wealth Quintiles	1	Referent		Referent	
	2	1.26 (0.95–1.67)	0.116	1.88 (1.54–2.31)	<0.001
	3	2.92 (2.27–3.76)	<0.001	3.10 (2.55–3.75)	<0.001
	4	6.56 (5.20–8.28)	<0.001	5.90 (4.91–7.10)	<0.001
	5	5.12 (4.05–6.49)	<0.001	8.77 (7.26–10.60)	<0.001

* n = 778 which is the sample with measured cholesterol

Out of the total 20.6% (423) of the people who required treatment according to WHO PEN guidelines, only 68 (15.8%) were receiving the required treatment (Table 3), however, our survey didn’t ask about ACE inhibitor use, so we were unable to report on this. Of the 431 (52.3%) people who met the criteria for either diagnosis of hypertension, diabetes and/or dyslipidaemia 84 (19.3%) were on any treatment.

Factors associated with being on treatment if recommended using either a WHO-PEN based or treat to target approach are presented in Table 4. In general, living in an urban area, being female, being older than 50 years of age, educated, being single/married/divorced, or in a higher wealth quintile, was associated with being on treatment when required.

### Readiness to provide care

For the SARA survey, a total of 138 facilities were included, the majority were primary care facilities (94.2%) and the remaining were secondary care facilities. Of primary care facilities, 73.1% were found in the rural areas and 87.5% of secondary care facilities were in urban areas (Appendix 3 in S1 File). 92% of all facilities were government-owned and almost all of these were primary care facilities. Only 12.5% of secondary facilities were government-owned. Considering general amenities, most facilities didn’t have their own power supply and just over half had a water supply (55.8%). 62.3% of facilities–all of them primary care—operated outpatient services only. Most facilities had a private consultation room (84.8%), reported access to emergency transportation (68.1%), and communication equipment (77.5%).

The overall readiness scores for CVDRF care for all facilities in Bo district was 16.8% (Table 5 and Appendix 4 in S1 File). Domain score for basic equipment was over half (67.0%), but only 5.8% of facilities had any glucometers. Diagnostic capacity score was low (8.8%) with very few facilities having any of the required diagnostic equipment [12]. Only two facilities had an ECG, none of the secondary care facilities had a CT scanner, and just 2.2% of all facilities had trained staff for CVDRF care. The medicines domain scored 5.9% with fewer than 10% of all facilities having each required medication, apart from aspirin (23.2% of facilities). None of the facilities had guidelines for diagnosis and management of CVDRF. Overall readiness score for HIV care in all facilities was 41.0% (Table 5 and Appendix 5 in S1 File). Basic equipment domain score was 80.2% and diagnostic capacity had the lowest score (18.6%). Trained staff for HIV care and availability of medicines for HIV scored 41.3% and 45.0% respectively. Guideline score was low (19.9%).

**Table 5:** Summary readiness scores for cardiovascular disease and cardiovascular disease risk factors (CVDRF) and HIV/AIDS for selected secondary and primary care facilities in Bo, Sierra Leone–see S1 File for components in each domain.

	CVDRF			HIV/AIDS		
	Secondary care (n = 8)	Primary care (n = 130)	All (n = 138)	Secondary care (n = 8)	Primary care (n = 130)	All (n = 138)
**Overall score**	44	15.2*	16.8*	52.8	40.3*	41*
**Domain**	**Domain score (%)**	**Domain score (%)**	**Domain score (%)**	**Domain score (%)**	**Domain score (%)**	**Domain score (%)**
**BASIC EQUIPMENT**	85	65.8	67	91.7	79.5	80.2
**DIAGNOSTIC CAPACITY**	55	8	10.9	25	18.2	18.6
**STAFF**	25	0.80	2.20	56.3	40.4	41.3
**MEDICINES**	50	2.9	5.88	53.8	44.6	45
**GUIDELINES**	0	0	0	37.5	18.8	19.9

*p<0.05

Comparisons were done between overall CVD and HIV readiness. Differences were statistically significant for all facilities and also primary care facilities.

There was a statistically significant difference between overall readiness scores obtained for CVDRF and HIV for all facilities (p = 0.018); and for primary facilities (p = 0.017). Difference between readiness scores for CVDRF and HIV at secondary facilities were not statistically significant (p = 0.550).

## Discussion

In this study we have triangulated across multiple data sources to show the need for and state of access to care for CVDRF among people 40 years of age or above living in Bo, Sierra Leone. We have shown that a moderate proportion of the population has a high WHO-ISH 10-year risk score for CVD. Moreover, a substantial proportion requires treatment based on risk-based WHO PEN guidelines or diagnostic criteria for individual CVDRFs. Receipt of care for those requiring treatment is low, regardless of whether based upon presence of individual risk factors or need for treatment based upon WHO PEN guidelines. We also found that facilities were not ready to manage CVDRFs, the readiness was especially low in primary care facilities, and there were differences between readiness to provide CVDRF care compared HIV/AIDS care at facilities.

The prevalence of CVDRFs is increasing in low income countries (LIC), and our findings add valuable knowledge of the high rates of CVDRFs and low access to care for these in Sierra Leone [2, 36–39]. Similar issues have been shown in neighbouring countries in Sub-Saharan Africa [35, 39–43]. Although the majority of our study population did not have high overall CVD risk according to WHO-ISH risk prediction, the percentage of people in the high risk category (>20%) is still large and greater than what has been shown in other LMICs [44]. One of the factors associated with having increased risk was living in an urban area, which could indicate that continuing increase in urbanisation might give higher WHO risk among the Sierra Leonean population in the future, as has been seen in upper middle income-countries such China [45, 46]. However other studies from other LMICs have suggested CVDRFs might have reached their peak in urban areas [46, 47]. Other factors associated with increased risk in our study population were low education level, or being unmarried, showing an unequitable distribution of risk, as concurs with other studies in LICs [44, 45]. Our results that show that more of the population require treatment for individual risk factors using a treat-to target approach than when using risk-based guidelines (we used WHO PEN) concurs with other studies, some of which have additionally demonstrated large cost-benefit gains in using risk-based treatment [14].

We have also shown that many people are without access to the treatment that they need to prevent future cardiovascular disease, with met need for care being low regardless of whether need was assessed based on presence of individual risk factors or overall risk. However, met need for care was lower when considering the WHO PEN risk-based approach than a treat-to-target approach, which perhaps reflects a low uptake of the risk-based approach amongst care-providers. In our study population, people were more likely to access care if they lived in an urban area, were of female gender, were in the age group 50–59, had education, or had higher wealth. Hence access to care is inequitable. This has previously been shown in other settings when assessing equitability of progressing through the cascade of care for CVDRF, suggesting that equitable access to care is a universal problem and priority needs to be given to those who are known to be at high risk, but have low levels of access [3, 9]. Along with poor met need for care, we have shown that the burden of CVDRF is not matched by readiness of the health services to deal these conditions. We found there were inadequacies in all of the domains for health services readiness for CVDRFs that were assessed in the SARA survey, with staff and medicines domains remarkably poor. Developing health services to enable equitable access for people who need to treat cardiovascular disease risk factors is complex, and no one factor can improve access [9]. However, key requirements are political will and funding [20, 48]. Whilst Sierra Leone has a NCD plan and a director of NCDs in the MoHS, funding for NCDs is clearly inadequate [49]. Of the total ODA budget for Sierra Leone, only 510,000$ were earmarked for all NCD care in 2017 [21]. Considering a major part of the general health budget in Sierra Leone comes from external funding, international aid funders need to give considerably more attention to NCDs and CVDRFs, which may potentially overload the health system in the coming years.

We compared readiness for CVDRF with that for HIV, considering HIV as an exemplar of what could be achieved with sustained funding and political attention. HIV was a leading health focus of the Millennium Development Goals and Sierra Leone receives around $30 million per year in funding [20, 21]. We found that although preparedness for HIV was not high, there was significantly higher preparedness for HIV care compared to CVD care in primary care facilities, indicating that similar achievements could be seen for CVDRF in Sierra Leone if there is commensurate funding and political will. Although our aim was not to prioritise one health area over another, especially given the different disease trajectories of HIV and CVDRF, it is worth reflecting that prevalence of HIV in Sierra Leone is 1.7% [50], whereas CVDRFs have been shown to affect up to 77% of the population. This difference in need suggests that funding and political attention for CVDRF could and should be increased [2].

This study has several limitations. First of all we could only measure cholesterol in half of the sample due to lack of resources, and therefore some of the analyses had to be done without including hyperlipidaemia. However as we have shown in Table 1 there were few differences between the participants with measured cholesterol and those without measured cholesterol. In addition the WHO-ISH risk prediction algorithm allows for missing cholesterol data, increasing its feasibility in low resource settings, and allowing an estimate of CVD risk to be calculated for the full sample in this study. We didn’t account for individual’s knowledge of disease status when assessing whether treatment was given for CVDRF. Although knowledge of a disease is a pre-requisite for treating it, our assessment started from the assumption that all those who needed treatment should be on it. The data collection for the household survey was also limited to areas within 40 km of Bo City due to accessibility. However all chiefdoms within Bo District are within this radius and were included and randomised; it is unlikely that the areas further away from Bo would be different to ones selected in this study. We did not control for clustering at household level as few houses supplied more than one participant. Moreover participants in our study sample could be accessing care, but not be on medications due to costs, poor technical quality and stockouts.

## Conclusion

In this study we have shown multiple interconnecting factors related to low met care-need for CVDRF in Bo, Sierra Leone. The prevalence of CVDRFs is high and a moderate proportion of the study population is at high risk for future cardiovascular events. A large proportion of the study population should be under primary prevention treatment according to WHO PEN guidelines, but very few currently are. The number of people needing treatment is even higher when using individual diagnostic cut offs for CVDRFs. The health system preparedness for treating CVDRFs and CVD events is remarkably low and significantly lower than for HIV care. Our results suggest multiple ways of improving met-need for care, including prioritising initiatives to increase access in those groups whose access to care is significantly low. However, all initiatives require funding, and the notably low levels of funding for CVDRFs and NCDs requires to be addressed if Sierra Leone is going to be able to achieve equitable access to quality care for CVDRF in the near future.

## Supporting information

S1 File (PDF)
