# Supplementary material for: Cardiovascular disease risk profile and management among people 40 years of age and above in Bo, Sierra Leone: A cross-sectional study
Source: PLoS One. 2022 Sep 9;17(9):e0274242. doi: 10.1371/journal.pone.0274242 (PMC9462708; doi:10.1371/journal.pone.0274242)
Supplement: S1 File — (PDF) [file pone.0274242.s001.pdf]

Appendix 1: Domains included in SARA survey scores

Domain	Cardiovascular items/Component
BASIC EQUIPMENT	Blood pressure apparatus
	Glucometer
	Stethoscope
	Weighing scale
	Measuring tape/height board
DIAGNOSTIC CAPACITY	Blood glucose
	Urine dipstick glucose
	Urine dipstick protein
	ECG
	CT Scan
STAFF	Trained staff for diagnosis and management of CVD/CVDRF
MEDICINES	Beta-blockers
	ACE-Inhibitors
	Thiazide diuretics

	Insulin
	Glicazide
	Glibenclamide
	Metformin
	Aspirin
	Simvastatin/other statins
GUIDELINES	Guidelines for diagnosis and management of CVD/CVDRF

Appendix 2: WHO PEN guidelines for treatment of cardiovascular disease risk factors

**Antihypertensive**	**Anti-hyperglycaemic**	**Statin**	**ACE I**	**Aspirin**
BP ≥160/100	Diabetes defined as above	Diabetes diagnosis and 10-year CVD risk >20%	Diabetes diagnosis and 10-year CVD risk >20%	Diabetes diagnosis and 10-year CVD risk >20%
BP ≥140/90 and 10-year CVD risk >20%		CVD risk >30%		CVD risk >30%

Appendix 3: Descriptive table for facilities in Bo district

Variables	GROUPS	N (%)
**FACILITY TYPE**	All	138
	Secondary care hospitals	8 (5.8)
	Primary care clinics	130 (94.2)
**LOCATION**		
Rural	All	96 (69.6)
	Secondary care facilities	1 (12.5)
	Primary care facilities	95 (73.1)
Urban	All	42 (30.4)
	Secondary care facilities	7 (87.5)
	Primary care facilities	35 (26.9)
**OWNERSHIP**		
Government	All	127 (92.0)
	Secondary care facilities	1 (12.5)
	Primary care facilities	126 (96.9)
Private	All	11 (8.0)
	Secondary care facilities	7 (87.5)
	Primary care facilities	4 (3.1)
**AMENITIES BY FACILITY TYPE**		
Power supply	All	23(16.7)
	Secondary care hospitals	5 (62.5)
	Primary care clinics	18 (13.8)
Improved Water source	All	77 (55.8)
	Secondary care hospitals	7 (87.5)
	Primary care clinics	70 (53.8)
Outpatient only	All	86 (62.3)
	Secondary care hospitals	0
	Primary care clinics	86 (100)
Private consultation room	All	117 (84.8)
	Secondary care hospitals	8 (100)
	Primary care clinics	109 (83.8)
Emergency transport	All	94 (68.1)
	Secondary care hospitals	6 (75.0)

	Primary care clinics	88 (67.7)
Communication equipment	All	107 (77.5)
	Secondary care hospitals	7 (87.5)
	Primary care clinics	100 (76.9)

Appendix 4: Readiness score for cardiovascular disease and cardiovascular disease risk factors among selected secondary and primary care facilities in Bo, Sierra Leone

	Secondary care (n= 8)			Primary care (n=130)		All (n=138)	
**Overall score**			**44**		**15.2**		**16.8**
Domain	Cardiovascular items/Component	Items present{Yes - n (% of facilities)}	**Domain score (%)**	Items present{Yes - n (% of facilities)}	**Domain score (%)**	Items present{Yes - n (% of facilities)}	**Domain score (%)**
BASIC EQUIPMENT	Blood pressure apparatus	7 (87.5)	**85%**	102 (78.5)	**65.80%**	109 (79.0)	**67%**
	Glucometer	6 (75.0)		2 (1.5)		8 (5.8)	
	Stethoscope	7 (87.5)		119 (91.5)		126 (91.3)	
	Weighing scale	8 (100)		80 (61.5)		88 (63.8)	
	Measuring tape/height board	6 (75.0)		125 (96.2)		131 (94.9)	
DIAGNOSTIC CAPACITY	Blood glucose	6 (75.0)	**55%**	2 (1.5)	**8%**	8 (5.8)	**10.90%**
	Urine dipstick glucose	7 (87.5)		17 (13.1)		24 (17.4)	

	Urine dipstick protein	7 (87.5)		20 (15.4)		27 (19.6)	
	ECG	2 (25)		0		2 (1.4)	
	CT Scan	0		NA		0	
STAFF	Trained staff for diagnosis and management of CVD/CVDRF	2 (25.0)	**25%**	1 (0.8)	**0.80%**	3 (2.2)	**2.20%**
MEDICINES	Beta-blockers	5 (62.5)	**50%**	1 (0.8)	**2.90%**	6 (4.3)	**5.88%**
	ACE-Inhibitors	5 (62.5)		0		5 (3.6)	
	Thiazide diuretics	6 (75.0)		1 (0.8)		7 (5.1)	
	Insulin	2 (25.0)		2 (1.5)		4 (2.9)	
	Glicazide	2 (25.0)		0		2 (1.4)	
	Glibenclamide	4 (50.0)		0		4 (2.9)	
	Metformin	3 (37.5)		6 (4.6)		9 (6.5)	
	Aspirin	8 (100)		24 (18.5)		32 (23.2)	

	Simvastatin/other statins	1 (12.5)		3 (2.3)		4 (2.9)	
GUIDELINES	Guidelines for diagnosis and management of CVD/CVDRF	0	**0%**	0	**0%**	0	

Appendix 5: Readiness score for HIV/AIDS care among selected secondary and primary health care facilities in Bo, Sierra Leone

Facilities (N)	SECONDARY (8)				PRIMARY(130)				ALL (138)			
Domain	HIV items/components	Items present {Yes} N(%)	Domain score (%)	Overall readiness score (%)	HIV items/components	Items present{Yes} N(%)	Domain score (%)	Overall readiness score (%)	HIV items/components	Items present {Yes} N(%)	Domain score (%)	Overall readiness score (%)
	Weighing scale	8 (100)			Weighing scale	80 (61.5)			Weighing scale	88 (63.8)		
Basic equipment	Thermometer	7 (87.5)	91.7		Thermometer	111 (85.4)	79.5		Thermometer	118 (85.5)	80.2	
	Stethoscope	7 (87.5)			Stethoscope	119 (91.5)			Stethoscope	126 (91.3)		
	HIV test kits	6 (75.0)		52.8	HIV test kits	69 (53.1)		40.3	HIV test kits	75(54.3)		41
Diagnostic capacity	Complete blood count (CBC)	0	25		Complete blood count (CBC)	1 (0.8)	18.2		Complete blood count (CBC)	1 (0.7)	18.6	
	CD4 or viral load	0			CD4 or viral load	1 (0.8)			CD4 or viral load	1 (0.7)		

Staff	Trained staff for diagnosis and management of HIV	4 (50)	56.3		Trained staff for diagnosis and management of HIV	45 (34.6)	40.4		Trained staff for diagnosis and management of HIV	49 (35.5)	41.3	
	Trained staff for counselling and HIV testing	5 (62.5)			Trained staff for counselling and HIV testing	60 (46.2)			Trained staff for counselling and HIV testing	65 (47.1)		
Medicine	Three first line ARTs	3 (37.5)	53.8		Three first line ARTs	50 (38.5)	44.6		Three first line ARTs	53 (38.4)	45	
	Cotrimoxazole/ other(for primary prevention of opportunistic infections)	7 (87.5)			Cotrimoxazole/other (for primary prevention of opportunistic infections)	108 (83.1)			Cotrimoxazole/ other(for primary prevention of opportunistic infections)	115 (83.3)		

	analgesics (for palliative care)	8 (100)			analgesics (for palliative care)	129 (99.2)			analgesics (for palliative care)	137 (99.3)		
	fortified protein supplementation (FSP)	2 (25)			fortified protein suppleme ntation (FSP)	25 (19.2)			fortified protein supplementation (FSP)	27 (19.6)		
	vitamins/Iron/any micronutrient supplements	4 (50)			vitamins/I ron/any micronutri ent suppleme nts	55 (42.3)			vitamins/Iron/any micronutrient supplements	59 (42.8)		
	TB preventive treatment	4 (50)			TB preventive treatment	11 (8.5)			TB preventive treatment	15 (10.9)		
	Anti-fungals	3 (37.5)			Anti-fungals	27 (20.8)			Anti-fungals	30 (21.7)		
	other treatment for opportunistic infections	4 (50)			other treatment for opportunis tic infections	53 (40.8)			other treatment for opportunistic infections	57 (41.3)		

	ARV prophylaxis for HIV+ pregnant women	4 (50)			ARV prophylaxis for HIV+ pregnant women	61 (46.9)			ARV prophylaxis for HIV+ pregnant women	65 (47.1)		
	Prophylaxis for newborns of HIV+ mothers	4 (50)			Prophylaxis for newborns of HIV+ mothers	59 (45.4)			Prophylaxis for newborns of HIV+ mothers	63 (45.7)		
Guidelines	Diagnosis and management guidelines for HIV	2 (25)	37.5		Diagnosis and management guidelines for HIV	17 (13.1)	18.8		Diagnosis and management guidelines for HIV	19 (13.8)	19.9	
	Guidelines for counselling and testing	4 (50)			Guidelines for counselling and testing	32 (24.6)			Guidelines for counselling and testing	36 (26.1)		
